# Compo: composite motif discovery using discrete models

**DOI:** 10.1186/1471-2105-9-527

**Published:** 2008-12-08

**Authors:** Geir Kjetil Sandve, Osman Abul, Finn Drabløs

**Affiliations:** 1 Department of Computer and Information Science, Norwegian University of Science and Technology (NTNU), Trondheim, Norway; 2 Department of Computer Engineering, TOBB University of Economics and Technology, Ankara, Turkey; 3 Department of Cancer Research and Molecular Medicine, Norwegian University of Science and Technology (NTNU), Trondheim, Norway; 4 Department of Informatics, University of Oslo, Norway

## Abstract

**Background:**

Computational discovery of motifs in biomolecular sequences is an established field, with applications both in the discovery of functional sites in proteins and regulatory sites in DNA. In recent years there has been increased attention towards the discovery of composite motifs, typically occurring in cis-regulatory regions of genes.

**Results:**

This paper describes Compo: a discrete approach to composite motif discovery that supports richer modeling of composite motifs and a more realistic background model compared to previous methods. Furthermore, multiple parameter and threshold settings are tested automatically, and the most interesting motifs across settings are selected. This avoids reliance on single hard thresholds, which has been a weakness of previous discrete methods. Comparison of motifs across parameter settings is made possible by the use of p-values as a general significance measure. Compo can either return an ordered list of motifs, ranked according to the general significance measure, or a Pareto front corresponding to a multi-objective evaluation on sensitivity, specificity and spatial clustering.

**Conclusion:**

Compo performs very competitively compared to several existing methods on a collection of benchmark data sets. These benchmarks include a recently published, large benchmark suite where the use of support across sequences allows Compo to correctly identify binding sites even when the relevant PWMs are mixed with a large number of noise PWMs. Furthermore, the possibility of parameter-free running offers high usability, the support for multi-objective evaluation allows a rich view of potential regulators, and the discrete model allows flexibility in modeling and interpretation of motifs.

## Background

Computational discovery of motifs corresponding to functional sites in proteins or binding sites in DNA is an established field within bioinformatics. In particular, the discovery of transcription factor binding sites in DNA has received much attention. Experimental identification of binding sites is a tedious process. Given the ever increasing number of genomes that are sequenced, computational identification of regulatory elements is needed to speed up the annotation process.

A typical approach for motif discovery is to use regulatory (promoter) regions for genes that are believed to be co-regulated as input, and try to predict individual DNA binding sites and possibly associated transcription factors that can explain the co-regulation. Typical software tools are MEME [1] and AlignACE [2]. This has turned out to be a very challenging problem. In particular the large number of false positive binding sites predicted by most methods represents a problem [3]. One promising improvement to this strategy is to search for combinations of binding sites, rather than individual occurrences.

Gene regulation usually has a combinatorial complexity [4], *i.e*. a combination of transcription factors (TFs) is often needed for active regulation. These TFs may be co-acting either directly through physical contact or indirectly through additional factors. As co-acting TFs may be expected to be in physical proximity, their binding sites are often clustered in sequence space. However, this is not a strict requirement as the DNA strand may form loops between distant sites [5]. Also, a given regulatory region may contain several possibly independent subsets of TF binding sites, representing alternative regulatory contexts. Clusters of binding sites involved in co-regulation are often referred to as cis-*regulatory modules *(CRMs), *composite motifs *or *structured motifs *[6], and they usually contain binding sites for a few TFs [7]. In this paper we refer to the model of a binding site of an individual TF as a *single motif*, and a given set of single motifs as a *composite motif*. We also use the term *module *when we want to emphasize the biological aspects of the TF combination.

Several computational methods have been developed for the discovery of composite motifs [8]. One line of methods, often called *de novo *module discovery, tries to find composite motifs using only DNA sequences as input data (e.g. CisModule [9], LOGOS [10], EMCMODULE [11]). This is a notoriously difficult problem, in many cases with close to random performance. However, biologists will often have some prior knowledge about potential regulators for the sequences of interest. Therefore another line of methods takes a list of single motifs as input along with the sequence data (e.g. Cister [12], ModuleSearcher [7], MScan [13]). These methods can also be used in a *de novo *setting by first finding candidate motifs using a single motif discovery method, and then running composite motif discovery with the candidate motifs as input. The differences between composite motif discovery methods lie mainly in 1) how single motifs and inter-motif distance conservation are modeled, 2) how motifs are evaluated and ranked, and 3) how the search space of composite motifs is explored. Composite motifs can be modeled in a discrete or probabilistic framework. Discrete methods typically use a set-model for composite motifs, requiring all single motifs to occur in a composite motif instance [14,15]. This is typically combined with a discrete model for inter-motif distance restriction, the most common approach being a window model that requires all motifs to occur within a sequence window of given length, but without any constraints on internal order or distances between single motifs (e.g. [7,16]). The discrete approach has several advantages, such as efficient inference and straightforward interpretation of motifs, and often an exhaustive mapping of the search space is possible. However, the reliance on hard thresholds for discretization may pose problems because of uncertainty and variability of TF binding. Therefore, the recent trend has been towards probabilistic models of composite motifs. Hidden Markov Models (HMMs) have often been used [10,12,17], typically containing different states for each single motif as well as for intra- and inter-module gaps. However, given the advantages of discrete methods we believe that they still can be a useful supplement and alternative to probabilistic methods.

This paper describes a new method Compo, which revisits the discrete approach to composite motif discovery. Compo relaxes the limitation of hard discretization thresholds by using multiple threshold values. By using p-values as a general significance measure, comparison of motifs across threshold settings becomes possible and thus automatic selection of the most interesting motifs across several threshold values. Furthermore, the automatic selection across parameter values means that Compo is able to infer properties of composite motif structure. Compo is therefore able to exploit overrepresentation across co-regulated sequences for improved composite motif detection. Although parameter inference from data is also possible with models such as HMMs, most proposed methods only scan HMMs against target sequence, using fixed parameters for module structure (e.g. [12,17,18]). This is basically equivalent to a single-sequence approach. Compo supports a richer composite motif model than previous discrete methods.

In addition to the standard set-model of component motifs, it optionally allows some component motifs to be missing (fault-tolerance) in composite instances, and distance restrictions on composite instances can optionally be enforced. As motif significance is computed as p-values for all supported models, the significance of composite motifs having different structure can easily be compared. An improved background model is also introduced, which combines empirical scanning against real background DNA at the single motif level with model based computations at the composite level. Compo can return either an ordered list of motifs, ranked according to p-values, or a Pareto front (solution set containing solutions not dominated in at least one dimension of objectives) corresponding to a multi-objective evaluation with sensitivity, specificity and spatial clustering as independent objectives. The multi-objective approach gives a collection of resulting motifs displaying more varying characteristics, and it allows necessary trade-offs between objectives to be made while analyzing the results, rather than prior to running the method.

## Results

Here we present the Compo algorithm for motif discovery by first introducing the necessary definitions and specifying the relevant problems. We then give the practical implementation of the algorithm. Finally we present the experimental evaluation of the implementation.

### Definitions

Let *S *= {*S*_1_, ..., *S*_*i*_, ..., *S*_*n*_} be a set of *n *symbol sequences each of which is defined over the alphabet Σ; for DNA sequences Σ = {*A*, *C*, *G*, *T*}. Let *M *= {*M*_1_, ..., *M*_*j*_, ..., *M*_*m*_} be a set of *m *motifs of interest. We assume that for each sequence – motif combination there exists a specific function which gives start positions for all instances of the motif on the sequence; i.e., a function Φ : Σ* × *M *→ 2^{1,2,...,|Σ*|}^.

**Definition 1 (Motif Support) ***Given the function *Φ, *sequence S*_*i *_*is said to *support *motif M*_*j*_, *denoted *$SS_{S_{i}}$(*M*_*j*_), *if *Φ(*S*_*i*_, *M*_*j*_) ≠ ∅. *Moreover, the *support set *of M*_*j *_*is all the sequences in S that support M*_*j*_; *i.e. SS*_*S*_(*M*_*j*_) = {*S*_*i*_|*S*_*i *_∈ *S *∧ Φ(*S*_*i*_, *M*_*j*_) ≠ ∅}. *The *absolute support *is then the size of SS*_*S*_(*M*_*j*_), *i.e*. |*SS*_*S*_(*M*_*j*_)|.

**Definition 2 (Module Support) ***Given the function *Φ, *sequence S*_*i *_∈ *S is said to *support *module M*^*s *^⊆ *M*, *denoted *$SS_{S_{i}}$(*M*^*s*^), *iff *∀*M*_*j *_∈ *M*^*s *^Φ(*S*_*i*_, *M*_*j*_) ≠ ∅. *Moreover, the *support set *of M*^*s *^*is all the sequences in S that support M*^*s*^; *i.e. SS*_*S*_(*M*^*s*^) = {*S*_*i*_|*S*_*i *_∈ *S *∧ ∀*M*_*j *_∈ *M*^*s *^Φ(*S*_*i*_, *M*_*j*_) ≠ ∅}. *The *absolute support *is then the size of SS*_*S*_(*M*^*s*^), *i.e*. |*SS*_*S*_(*M*^*s*^)|. *Note that *$SS_{S_{i}}$(*M*^*s*^) *is an indicator variable but SS*_*S*_(*M*^*s*^) *is a set of sequences*.

**Proposition 1 (Monotonicity of module support) ***Given any S, and any M*^*t *^⊆ *M*^*s *^⊆ *M*, *then SS*_*S*_(*M*^*s*^) ⊆ *SS*_*S*_(*M*^*t*^).

Interesting modules are modules supported by many of the sequences in *S*. This notion is formally defined as follows.

**Definition 3 (Frequent Module) ***For a given support threshold σ *∈ {1,2...,|*S*|}, *module M*^*s *^*is said to be frequent in S iff *|*SS*_*S*_(*M*^*s*^)| ≥ *σ*.

The useful metric of *support *is a set metric, i.e. defined over the sequence set *S*. On the other hand, given a single sequence *seq *∈ Σ*, it is also relevant to ask how likely it is that a given module has a hit in the sequence. We call the relevant metric *module hit-probability *which is formally defined next.

**Definition 4 (Module Hit-probability) ***Given a sequence seq *∈ Σ*, *hit-probability of module M*^*s *^⊆ *M is probability of seq supports M*^*s*^. *Formally, the *module hit-probability *of M*^*s *^⊆ *M is Prob*(*SS*_*seq*_(*M*^*s*^)).

**Proposition 2 (Monotonicity of hit-probability) ***Given any S, and any M*^*t *^⊆ *M*^*s *^⊆ *M*, *then Prob*(*SS*_*seq*_(*M*^*s*^)) ≤ *Prob*(*SS*_*seq*_(*M*^*t*^)).

The hit-probability can be virtually defined over arbitrary sequences. In this work, we are particularly interested in representative background sequences or sequences generated from the background model *BM*. For this reason, lower hit-probabilities correspond to higher specificity (divergence from the background).

**Definition 5 (Specific Module) ***Given a representative background sequence bgseq *~ *BM, and specificity threshold ψ, module M*^*s *^⊆ *M is called specific module iff Prob*(*SS*_*bgseq*_(*M*^*s*^)) ≤ *ψ*.

In addition to support and hit-probability, an important metric is the statistical significance (significance for short) defined below. Significance is interpreted as how improbable the observed support is in a corresponding set of background sequences.

**Definition 6 (Module Significance) ***Given S and BM, significance of module M*^*s *^⊆ *M is probability of having support of at least *|*SS*_*S*_(*M*^*s*^)| *in a background sequence set BS which is generated from BM and structurally equivalent to S, i.e*. |*S*| = |*BS*| *and *∀*i *∈ {1,2, ..., |*S*|} (*S*_*i *_∈ *S*) ∧ (*BS*_*i *_∈ *BS*) ∧ (|*S*_*i*_| = |*BS*_*i*_|). *Formally, the *module significance *of M*^*s *^⊆ *M is Prob*(|*SS*_*BS*_(*M*^*s*^)| ≥ |*SS*_*S*_(*M*^*s*^)|).

**Definition 7 (Significant Module) ***For a given significance threshold θ *∈ [0..1], *module M*^*s *^⊆ *M is *significant *if Prob*(|*SS*_*BS*_(*M*^*s*^)| ≥ |*SS*_*S*_(*M*^*s*^)|) ≤ *θ*.

### Problem specification

We consider three basic problem specifications (Problems 1, 2 and 3) within the setting presented above.

**Problem 1 (Frequent and Specific Modules) ***For fixed S, BM, M, and given support threshold σ and specificity threshold ψ, find all modules M*^*s *^⊆ *M which are frequent and specific*.

Problem 1 is very similar to well established itemset and sequential itemset mining problems [19]. In these problems, the solution space typically grows very large and many solutions are usually not interesting. Therefore users are allowed to define their interest by specifying constraints. The user defined constraints are enforced by the mining system in order to focus the search on the interesting solutions only [20]. Moreover, certain classes of constraints (e.g. monotonicity) make the search efficient: this is done by pushing the constraints inside the mining process.

What is common to itemset and sequential itemset mining approaches is the generation of complete solutions; i.e. every solution (frequent and specific modules) satisfies the user specified threshold parameters and constraints. On the other hand, in motif discovery problems, incomplete solutions employing heuristic searches are usually preferred. These solutions are supposed to optimize some well-defined optimality criterion (e.g. support or hit-probability). However, there is usually more than one optimality criterion, thus making the problem a multi-objective optimization problem [21]. There are basically two different ways to approach this. One possibility is to define a scheme for combining the different optimality criterions into a single criterion, score every motif according to this combined criterion, and return a list of motifs ranked according to score. The scheme for combining criterions may be ad hoc, or it may for instance be based on an unexpectedness scheme with ranking of p-values as described in *Motif scoring*. Ranking according to a single criterion is easy to relate to for a user. It is thus advantageous for novice users, when several data sets are analyzed rapidly, or when an objective criteria for selection is needed, such as with automatic benchmarks. We define the combined-objective approach to solution space as follows:

**Problem 2 (Top-ranking Modules) ***Given the motif set M, module size c, a desired number n of composite motifs to be returned, and a score function f mapping composite motifs to scalar score values, find the n top-ranking modules according to the score function, i.e. M*^*s *^⊆ *M s.t*. |*M*^*s*^| = *c and f*(*M*^*s*^) >= *f*(*M*^*t*^) *for any non-returned motif M*^*t*^.

The other possibility is to fully treat motif discovery as a multi-objective optimization problem with each objective representing a separate dimension of optimality. One can then return the Pareto front of composite motifs. The Pareto front contains all non-dominated motifs, where dominated means that there exists another motif with equal or better score values for all objectives. As this selects motifs that score high in different dimensions of optimality, it may give a more varied collection of output motifs. For in-depth analysis of a data set this may give a richer picture of potential regulators. We define the multi-objective approach to solution space as follows:

**Problem 3 (Pareto-optimal Modules) ***Given the motif set M, module size c, find Pareto front of M, i.e. M*^*s *^⊆ *M s.t*. |*M*^*s*^| = *c and M*^*s *^*is non-dominated in specified dimensions*.

The definition given in Problem 3 is very general in the sense that any number of dimensions can be incorporated. For instance, support and hit-probability can be selected as dimensions.

Given the dimensions of interest, the input sequence set and the background model, a straightforward complete solution to Problem 2 or Problem 3 can be obtained as follows.

Generate every *M*^*s *^⊆ *M s.t*. |*M*^*s*^| = *c *and output any motif satisfying the criteria in Problem 2 or 3.

The number of subsets of *M *can grow exponentially, for instance when *c *≈ |*M*|/2, thus making the straightforward approach infeasible when |*M*| is large. Fortunately, though the motif set *M *can be large (i.e. hundreds of motifs, e.g. the full TRANSFAC database), most biological modules comprise at most several individual motifs. So, by bounding *c *with a relatively small constant (e.g. 4 or 5), the straightforward approach becomes feasible, as the number of such subsets grows polynomially. This observation allows us to exhaustively consider only modules with up to several constituent motifs. The straightforward approach may become unpractical when |*M*| is large even though *c *is fixed to at most several. As a realistic approach for solving Problem 2 or 3 efficiently we propose the Compo algorithm as described in the next section. The main advances are in exploiting monotonicities and using heuristics and approximations for efficient module discovery. This enables Compo to cope with large |*M*| (order of hundreds).

### The Compo algorithm

This section gives a general overview of the Compo algorithm. Details on each step of the algorithm are given under relevant subsections of *Implementation*, as indicated below.

**Figure 1:**
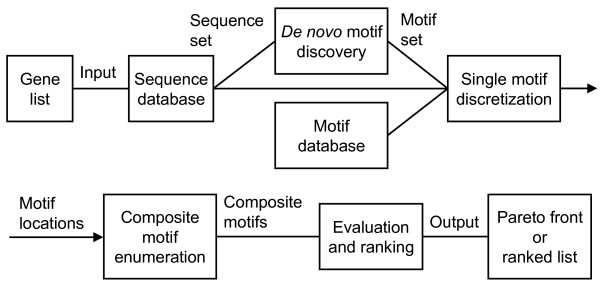
**Compo workflow**. The general workflow of Compo, from a list of genes defining regulatory regions of interest, to a Pareto front or ranked list of composite motifs as potential regulators of the genes.

The general workflow of Compo is shown schematically in Figure 1. A set *S *of regulatory regions is retrieved from a sequence database, and a set *M *of regulatory motifs is retrieved from a motif database or discovered *de novo *by any external method. The hit positions of all motifs *M*_*j *_∈ *M *in every sequence *S*_*i *_∈ *S *are then found (*Pre-processing of input*). Composite motifs are enumerated in an implicit search tree. For each enumerated composite motif node, the support and hit-probability are calculated. Support is the number of sequences with module hit; hit-probability is the (approximated) probability of having at least one module hit in a background sequence. For each node in the tree, these values are calculated from the values at the parent node and the values of the added single motif (*Enumeration of composite motifs*). Compo supports two alternative forms of output – a list of motifs ranked according to a combined significance measure (*Motif scoring*), or a Pareto front of optimal motifs according to a multi-objective optimization (*Pareto front*). Compo can optionally allow non-perfect matches (*Allowing non-perfect matches*) and enforce distance constraints (*Incorporating distance constraints*). Finally techniques used to make Compo as efficient as possible are briefly discussed (*Computational efficiency*).

### Implementation

#### Pre-processing of input

The first step of the analysis is determination of motif hits, i.e. the function Φ(*S*_*i*_, *M*_*j*_). As Compo operates on discretized motif hits, and thus works independently of the internal representation of single motifs, any external *de novo *motif discovery method or motif library can be used for this first step. If probabilistic motifs (e.g. PWMs) are to be used with Compo, the continuous match values at each position have to be discretized into hits and no-hits. This can be done by setting a hit threshold for each motif. Hit thresholds can be calculated algebraically or determined based on the resulting distribution of hits in input sequences and background sequences.

When the match values of motifs have a clear probabilistic interpretation, such as log-likelihoods or log-odds, it can be meaningful to simply set all hit thresholds to a universal, analytically reasoned value. Similarly, as p-values can be computed from match scores, hit-thresholds may be found that correspond to a specific p-value of motif match according to a stochastic sequence model. Alternatively, hit-thresholds may be set to control some property of the resulting hit-distribution, for example to achieve a specific frequency of hits in the input or background sequences. In general, any function can be defined on the number of hits in input and background sequences respectively, and the hit-threshold set to the value that optimizes this function.

In the current implementation we calculate a desired number of hits as the number of input sequences multiplied by a hit density factor, and then set the hit-threshold of each motif to the value that achieves this desired number of hits across the input sequences. For the initial step of obtaining continuous match values of motifs against sequences, we make use of the TAMO motif tools [22]. In the default setting, several values are tried for the hit density factor and the most significant motifs across density factor values are returned.

#### Enumeration of composite motifs

**Figure 2:**
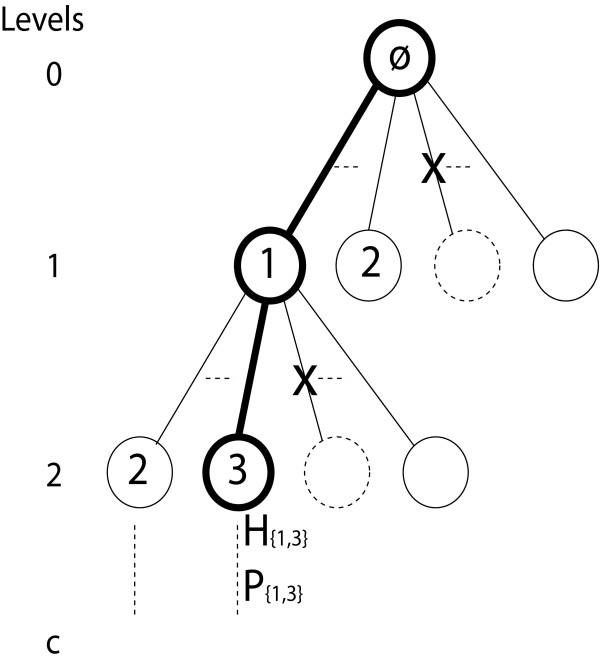
**Search tree**. Implicit search tree, where numbers inside nodes correspond to single motifs (*z*), and paths from the root to a node correspond to composite motifs. The values *H*_{1,3} _and *P*_{1,3} _corresponds to the path in bold. The X symbol indicates that some composite motifs will be pruned during search.

Combinations of single motifs are conceptually explored exhaustively in a search tree as shown in Figure 2. Each node (except the root) is associated with a single motif and each path from the root to a leaf node corresponds to a unique combination of single motifs (i.e. a composite motif). The number of levels in the search tree is constrained by a maximum number |*M*^*s*^| = *c *of motif components, given as a parameter to the algorithm. A search tree of *c *levels encompasses all combinations of up to *c *motifs. Each leaf or non-leaf node, *z*, with the respective single motif *M*_*z *_∈ *M*, has two basic variables associated with it: the support set *H*_*z *_= *SS*_*S*_(*M*_*z*_) and the hit-probability *P*_*z *_= *Prob*(*SS*_*bgseq*_(*M*_*z*_)). The values of *H*_*z *_and *P*_*z *_are pre-computed and used whenever needed. Additionally, each node has two other variables *H*_*X*.*z *_and *P*_*X*.*z *_for incrementally updating the partial module support and hit-probability, respectively. These values represent support and hit-probability of composite motifs represented by the path from root to node *z*. The *H*_*X*.*z *_and *P*_*X*.*z *_values for node *z *are calculated based on the accumulated values for parent node and *H*_*z *_and *P*_*z *_values, respectively.

The support set *H*_*X*.*z *_of module *X*.*z*, where *X *is the set of single motifs down to node *z*, can be computed from module support *H*_*X *_and single motif support *H*_*z *_by just intersecting the sets *H*_*X *_and *H*_*z*_. Formally, *H*_*X*.*z *_= *H*_*X *_∩ *H*_*z*_. The hit-probability *P*_*X*.*z *_can similarly be computed as *P*_*X*.*z *_= *P*_*X*_·*P*_*z*_. The root node of the search tree is an empty module, and as there are no single motifs that require match, *P*_*root *_is trivially 1, and *H*_*root *_is the set of all input sequences. Values for the nodes in the tree are then calculated incrementally down the tree in a depth-first order. This model was also considered in a previous paper [21].

#### Motif scoring

Compo can assign a score to each candidate composite motif and return a ranked list of composite motifs as output. This requires that several desirable characteristics, such as high support and low probability of hit in background, are combined into a single score value. We use an approximated p-value of observed composite motif support as our score measure. The generality of the p-value as a measure allows composite motifs with differing characteristics to be directly compared.

The significance of a composite motif, i.e. the approximated p-value of observed support, is computed by the following four steps:

1. Position-level probability: The probability that a single motif occurs at a specific location in a background sequence. This is estimated as the frequency of motif hits in real DNA sequences serving as background.

2. Sequence-level probability: The probability that a single motif occurs at least once in a sequence of given length. This is computed as the union of probabilities of occurring at any location. As an approximation, the match probabilities are assumed to be equal at all locations, ignoring auto-correlation. This gives the formula: 1 - (1 - *p*_*pos*_)^*l*^, where *l *is average length of sequences and *p*_*pos *_is the probability of motif hit at a single position from the background model *BM*.

3. Hit-probability (Composite motif-level probability): The probability that a composite motif is occuring in a sequence of given length. This is computed as the product of sequence probabilities of each motif component.

4. Significance p-value (Dataset-level probability): The probability of seeing at least the observed support in a corresponding set of background sequences. This is computed as the right tail of a binomial distribution, i.e. as the probability of obtaining at least *k *out of *n *successes with Bernoulli trial probability *p*. Here, *p *is the composite motif-level probability, *n *is the number of input sequences, and *k *is the support of the composite motif.

This scoring procedure is a mix of model-based (algebraic) and empirical evaluation of significance. A purely empirical evaluation would compute a p-value directly in point 4 by comparing observed support with support in several different background sets of sequences. Conversely, a purely algebraic evaluation would compute match probabilities in step 1 algebraically from probabilities at each motif position according to a simplified DNA model.

The empirical and algebraic approaches each have their strengths and weaknesses. As the motif score is used to contrast potential binding sites against surrounding DNA sequence, having a background that is as realistic as possible is desirable. DNA sequences have several properties that depart from random sequence models, and using the frequency of hits in real genomic sequence may thus capture the background more accurately. On the other hand, estimates based on empirical frequencies become inaccurate when the frequency is low, and are limited by the minimum frequency. As the p-values in step 4 of the scoring procedure are often extremely low, the observed support would have to be compared against a huge collection of background sequence sets. Also at the third step the probability values are often very low when there are many motif components.

The mixed solution we have chosen combines advantages of both approaches. As the position-level probability is estimated from hits in real DNA, we have avoided assuming a simplified model of genomic background sequences at the local level. Using algebraic computations at step 2 and 3 instead assumes a random (simplified) model of the spatial distribution of motif occurrences. It is of course possible that motifs are unevenly distributed even in the background model of non-modules, but we consider this assumption less problematic. As the values at step 3 and 4 are computed algebraically, they are not limited by the lowest possible empirical frequency. Also, efficient algebraic formulas are used for computing values at step 2, 3 and 4 in the large search space of composite motifs, while the computationally demanding process of scanning against real negative data in step 1 is only performed once for each single motif, in the initial phase of the analysis.

Our calculations in step 2 and 3 are based on simple and approximate formulas which ignores correlations. The main motivation for this approach is the efficiency of the simple and incremental calculation in the search tree, as described in *Enumeration of composite motifs*. Actually, similar tradeoffs for increased efficiency are inherent in most motif discovery methods [23] due to the difficulty of the problem.

#### Pareto front

As an alternative to motif ranking based on a combined score, Compo also supports motif discovery as a multi-objective optimization problem. The composite motif-level probability described in *Motif scoring *then constitutes an independent final objective. Composite motif support and enforced distance restriction form additional separate objectives, and a Pareto front of motifs is returned as described in *Problem specification*.

Intuitively, the Pareto front contains motifs that have at least one or a few very good characteristics. This may make the motif discovery process more informative, as the returned motifs typically represent a broader view of the composite motif space (i.e. motifs with more varied characteristics) compared to the same number of motifs from a list ranked according to a single combined objective. The Pareto front can be visualized as an n-dimensional heat map, allowing the user to get an overview of trends in the results. After the search is finished, the user may decide on how to balance different criteria against each other and inspect motifs with desired combination of properties.

#### Allowing non-perfect matches

It is in some cases biologically relevant to allow for occasional absence of individual TF binding sites in module instances. This may be a desirable feature even if we assume that the module always contains the full set of binding sites, as it makes the approach more robust against inaccuracies in the single motif scanning step.

In this case we need to know the number of allowed motif mismatches in order to determine hit-probability and the support set. We say that a composite motif is defined by its component motifs, and refer to the different possibilities of allowed number of lacking motif matches as variants of the composite motif. A variant allowing *q *mismatches for a composite module consisting of a set *X *of single motifs is denoted as $V_{X}^{q}$. When we want to isolate a particular component motif we write $V_{X.y}^{q}$, where *y *is the single motif of particular interest and *X *is the set of remaining single motifs. In order to compute values incrementally from the already computed values of parent and newly added single motif, we need to keep values for different numbers of allowed motif misses. More specifically, a variant $V_{X.y}^{q}$ generally uses the pre-computed values of two variants of the parent single motif, $V_{X}^{q}$ and $V_{X}^{q-1}$, as well as values of the additional motif *y*. In this case the support set and hit-probability is computed incrementally as follows, where *q *refers to the number of allowed mismatches.

$$H_{X.y}^{q}=H_{x}^{q-1}\cup (H_{X}^{q}\cap H_{y}^{0})$$

As hits for the new motif *y *are assumed independent from hits for the motifs *X *in background, and since $H_{X}^{q-1}$ is a strict subset of $H_{X}^{q}$, the formula for hit-probability becomes (detailed derivation given in Additional file 1):

$$P_{X.y}^{q}=P_{X}^{q-1}+(P_{X}^{q}-P_{X}^{q-1})\cdot P_{y}^{0}$$

In the case when the number of allowed mismatches is equal to or greater than the number of components, *H *is trivially the set of all sequences and *S *is 1. For each composite motif, values are computed for variants with 0...*q *mismatches (as long as *q *is not greater than the number of components).

#### Incorporating distance constraints

As co-acting TFs may be expected to be in physical proximity, their binding sites are often clustered in sequence space. This is not a strict requirement as the DNA strand may form loops between distant sites. However, in particular in combination with flexibility regarding single motif mismatches, which increases the general robustness of motif discovery, limits on motif distances can still be a reasonable assumption. Compo supports constraints on the distance between motifs by requiring component motifs to have hits within a sequence window of a specific length.

As each component motif may have several instances in a given sequence, it is not entirely trivial to check distance constraints. One possibility is to slide a window through a sorted list of all motif occurrences and check whether the sliding window at any point contains occurrences of all components. A second possibility is to enumerate all combinations of occurrences from each motif component and check whether all occurrences of any combination are within the window. We have as default chosen the second method, as it allows an intuitive recursive implementation and can easily be combined with options such as non-overlapping motifs and single motif mismatches.

Distance restrictions are also taken into consideration in motif scoring. Hit-probability is then the probability that the composite motif occurs within a distance window in a background sequence. As the composite motif may occur in any of the (overlapping) windows of the sequence, hit-probability is computed by combining the probability of occurring in the first window of the sequence, and the probabilities of occurring in any of the remaining windows given that it did not occur in the preceding window. The details on how distance constraints are enforced when computing the support set of a composite motif, and how hit-probability is computed, are given in Additional file 1.

#### Computational efficiency

The running time of Compo is mainly determined by the number of input sequences |*S*|, the number of input single motifs |*M*|, and the maximum number of motif components *c *considered. Several techniques are employed to increase the computational efficiency. While exploring the search space, motif values are computed incrementally down the tree from parent values and pre-computed active node values, instead of being computed from ground up each time. As the support sequences *H*_*X *_are computed as a set intersection, and the incremental computation of hit-probability is done algebraically, values at each node are computed with small computational effort. Furthermore, if there are many input sequences the computation of support set can be done very efficiently using bit strings. A branch-and-bound approach is used to prune the search tree. For each node visited in the tree, a bound on the highest achievable score for any node in the subtree is computed and compared against the Pareto front or ranked motif list discovered so far. If the bound is dominated by the current Pareto front or ranked list, the whole subtree is discarded from search space. For large runs more than 99.9% of the search tree is typically discarded this way. Details of the branch-and-bound approach are given in previous publication [21] and in Additional file 1.

### Testing

Compo was tested on a large benchmark suite [24] compiled from the TransCompel data base (v9.4) [25], in addition to two smaller suites compiled from muscle- [26] and liver-specific [27] genes, and a recent suite compiled from the REDfly database [28]. It was run with automatic parameter selection, meaning that for each data set Compo automatically selected parameter values from a list of discrete possibilities. Although the performance of Compo could have been further improved by manually specifying optimal parameter values, this could easily have caused overtuning and was therefore avoided.

In the main benchmark suite (compiled from the TransCompel database), target PWMs are mixed with randomly selected TRANSFAC PWMs that have no annotated binding sites in a given data set. These PWMs without annotated binding are referred to as noise PWMs, and are introduced to simulate a situation without accurate knowledge of the true regulators. The benchmark suite defines 6 different noise levels, where the percentage of noise PWMs varies between 0% and 99%. The highest noise level, denoted as 99%, uses the whole TRANSFAC as input and has thus really around 99.7% noise PWMs.

At each noise level, ten different data set versions are defined, corresponding to different random selections of noise PWMs. This benchmark thus defines a total of 600 runs on individual data sets, with each data set consisting of between 5 and 16 input sequences. The results on this benchmark are shown in Table 1. Compo outperforms all other methods on all noise levels of the benchmark.

**Table 1:** Prediction performance

Noise	Compo	CMA	ModuleSearcher	Stubb	MSCAN	MCAST	Cister	Cluster-Buster
No	**0.52**	0.34	0.33	0.17	0.29	0.39	0.23	0.28
50%	**0.49**	0.33	0.32	0.17	0.24	0.30	0.21	0.27
75%	**0.46**	0.31	0.32	0.15	0.07	0.22	0.16	0.20
90%	**0.41**	0.29	0.30	0.13	0.07	0.14	0.07	0.13
95%	**0.37**	0.27	0.31	0.09	0.04	0.09	0.03	0.08
99%	**0.35**	0.23	0.20	0.01	0.02	0.00	0.01	0.05

Prediction performance on the TransCompel benchmark data sets. The given scores are for the custom matrices version of the benchmark, with different levels of randomly selected matrices (noise) added to the data set. Score values equal to or better than Compo are shown in bold.

Given the good performance of Compo we further investigated how the performance was influenced by relevant unique features of Compo, in particular the background based on real DNA sequence and the possibility of inferring motif properties across co-regulated sequences. The partly empirical background computations are unique to Compo, while the possibility of inferring motif properties is shared with CMA and ModuleSearcher. Table 2 compares the default score of Compo with scores achieved when using only a random model of DNA (computations according to a multinomial sequence model instead of real background DNA) and when considering each sequence in isolation (and not support across several sequences). It seems that both the empirical background and the inference of composite motif properties across co-regulatory regions contribute strongly to the high performance of Compo. When either of these elements is removed, the performance of Compo drops to a level comparable to other methods on the TransCompel suite.

**Table 2:** Influence of background models and support

Compo setup	TransCompel, no noise	TransCompel, 50% noise
Default Compo	**0.52**	**0.49**
Random DNA model bg	0.36	0.35
Independent sequence runs	0.39	0.31

The table shows how prediction performance is influenced by using only a random DNA model in background computations (no real background DNA sequence), and by making predictions on sequences independently (no support).

On muscle and liver benchmarks the performance of Compo is equal to or better than most other methods, except for MSCAN on muscle data and Cluster-Buster on liver data (see Table 3). The benefit from support is less obvious here when judged by the nCC score. However, using support tends to give more conservative solutions with less false positives compared to independent sequence runs (data not shown). This benchmark also shows the effect of allowing non-perfect matches. The effect is most pronounced in the muscle data set where the relevant binding site motifs (Mef2, Myf, Sp1, SRF and TEF) on average are found in only 42% of the modules, compared to 57% for the liver data set and motifs (HNF-1, HNF-3, HNF-4 and CEBP).

**Table 3:** Results on muscle and liver data sets

Method	Muscle	Liver
Compo, independent sequence runs	**0.47**	**0.56**
Compo, support and allowing non-perfect matches	0.42	**0.57**
Compo, support and standard set-model	0.37	0.55
CMA	0.46	0.36
ModuleSearcher	0.46	0.43
Stubb	0.24	0.48
MSCAN	**0.50**	0.51
MCAST	0.30	0.50
Cister	0.36	0.31
Cluster-Buster	0.41	**0.59**

Prediction performance on the muscle and liver data sets. Score values equal to or better than main Compo run are shown in bold.

The benchmarks discussed above each have their strengths and limitations. The TransCompel benchmark is broad and robust, with 10 data sets, different levels of noise, and a total of 600 runs. However as TransCompel currently contains almost exclusively TFBS pairs, methods are only tested on the discovery of small composite motifs. The muscle and liver benchmark data sets have larger composite motifs, but with only 2 data sets and a total of 2 runs, the results are less robust. An interesting addition to these two benchmarks are presented in a recent article by Ivan et al. [28]. A total of 33 data sets were compiled based on data from the REDfly database [29]. The data sets from this benchmark have been made available, together with a relatively simple evaluation procedure. Performance data according to this evaluation procedure has also been made available for a few selected methods. The accompanying evaluation procedure requires exactly one composite motif instance to be predicted for each sequence, requires all predicted instances for a given data set to have equal length, and only evaluates the predictions of start locations, not length predictions of composite motif instances. Based on this, the sensitivity of predictions are calculated for each data set, along with a p-value of whether predictions are significantly better than random. The main performance measure is the number of data sets with significant prediction (at the 0.05 level).

We evaluated Compo on this benchmark according to the accompanying evaluation procedure that assumes CRM length of 750 bp for all data sets. Results are given in Table 4. Compo made significantly good predictions (at the 0.05 level) on 9 out of the 33 data sets. This is better than random and better than the methods CisModule (4) and MCD (4), similar to D2z (9) and Stubb (10), and lower than CSAM (14), the best performing method which was accompanying the benchmark.

**Table 4:** Results on Drosophila data sets

Method	#sign. results
Compo	**9**
CisModule	4
MCD	4
D2z	**9**
Stubb	**10**
CSAM	**14**

Prediction performance (the number of data sets with significant predictions at the 0.05 level) on the Drosophila data sets. There is a total of 33 data sets in the benchmark. Score values equal to or better than Compo are shown in bold. Results for other methods have been taken from Table 5 in supplementary material for [28]).

Further details on the experimental setup are given in Additional file 1.

#### Pareto front

Compo may optionally return a Pareto front corresponding to a multi-objective evaluation on sensitivity, specificity and spatial clustering. Intuitively, the Pareto front contains motifs that have good values for at least one or a few of these characteristics. This gives a broader view of possibly interesting motifs, and leaves the final selection of output motifs to a subjective evaluation by the user. In addition to giving a broad view, this also avoids combining different objectives by general formulas that are typically inferior to expert judgment. This defining property of multi-objective optimization, however, also means it is not suited for use in automatic benchmarking procedures. For this reason, we used a standard ranking of motifs according to a combined score in the benchmarks.

**Figure 3:**
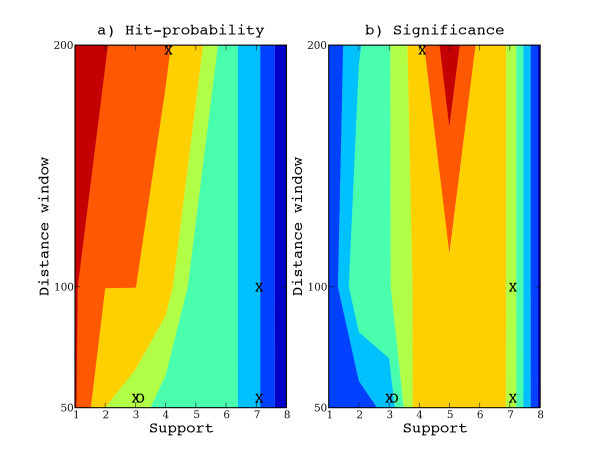
**Pareto front**. a) Pareto front of optimal composite motifs corresponding to a multi-objective optimization with respect to support, distance restriction and specificity (hit-probability). Red colors show high specificity and blue colors show low specificity. b) Corresponding layout of motifs where colors instead denote combined motif score (significance). Red colors correspond to highest-ranked motifs according to combined score. The top-ranked composite motif is located at support 5 and distance window 200.

To give an example of properties of Pareto fronts for composite motifs, we show the Pareto front for one of the data sets of the TransCompel benchmark presented above. On this data set, the highest-ranked motif predicted by Compo was not accurate. The top-ranking composite motif was composed of PWMs related to the Ets and GATA TFs, while the annotations for the data set specified a composite element composed of an AP1-related and a NFAT-related PWM. Figure 3a shows a heat map of the Pareto front for this data set, with support as first dimension, distance restriction as second dimension and specificity as third dimension (color). An interesting composite motif should typically have high support, be closely spaced, and be specific with respect to background. To the upper left are very specific (red) composite motifs with low support and low spatial clustering, while at the lower right are less specific (blue) with high support and high spatial clustering. Expert users may then make subjective judgements regarding trade-offs between these characteristics and further inspect composite motifs of interest.

Figure 3b shows a corresponding layout of the composite motifs on the x- and y-axes, but with the z-axis (color) representing the score of the composite motifs according to our combined score measure (p-values). This heat map show that a composite motif with support 5 and distance window 200 has the highest combined motif score (the composite motif composed of an Ets- and a GATA-PWM mentioned above). Some of the alternative composite motifs are composed of the true annotated TFs for this data set. Composite motifs with NFAT as component are marked with an X in the figure, while O denotes AP1. A composite motif with support 4 and a distance window of 200 is composed of both AP1 and NFAT. Although the highest-ranked composite motif was not related to any annotated TF, there are in the Pareto front other composite motifs with better spatial clustering (support 3, distance window 50) or better specificity denoted by orange color (at support 4, distance window 200) that contain one or both of the annotated TFs.

## Discussion

Given a set of genes (believed to be co-regulated), the objective with composite motif discovery methods is to predict transcription factors that are underlying regulators of the gene set. The starting point would be the gene list with known motifs for individual factors available from databases such as TRANSFAC [25] and Jaspar [30]. Alternatively, *de novo *single motif discovery may be performed to discover overrepresented short contiguous motifs in the sequences.

Given upstream gene regions and either known or *de novo *motifs, composite motif discovery methods such as Compo may be used to discover enriched combinations of motifs, which may correspond to cis-regulatory modules. With CC scores ranging from 0.35 to 0.52 on the TransCompel benchmark, Compo is consistently able to give useful computational binding site predictions for sets of co-regulated genes even when the true regulators are not known.

Users may have different levels of prior knowledge about the composite motifs they are seeking when they resort to a computational method. Some users may e.g. know the exact composition of the relevant module, whether all TFs are obligatory for the function of the composite motifs, and what the typical distances between binding sites in a module are. Other users may know nothing more than a list of TFs potentially regulating a list of genes. A computational method should therefore allow such intuitive parameters to be set if known, but it should not be necessary to set arbitrary values when no prior information is available. Compo allows many intuitive parameters to be set, but all of these parameters may also be estimated automatically. As Compo uses p-values as a universal significance measure, motifs discovered using different parameter settings can be directly compared. This allows Compo to be run with multiple settings and then automatically selecting the most significant motifs across these settings. By default Compo tries a large range of values for the number of components in modules; the size of the distance window, the allowed number of component motif misses, and the hit density factor used to determine hit-thresholds in the initial discretization. Furthermore, Compo has no so called nuisance parameters – parameters that reflect properties of the algorithm rather than properties of the module to be discovered.

The composite motif discovery method that is most similar to Compo is probably ModuleSearcher [7]. However, although Compo and ModuleSearcher are similar in search algorithm, there are also important differences. Compo uses real background DNA in its score computations, and may instead of a ranked list also return a multi-objective solution as output. If a standard ranked list is chosen as output in Compo, the composite motifs are ranked by p-values, which also allows composite motifs to be compared across parameter settings. Instead of relying on a fixed or specified value for each parameter, Compo can thus take a list of candidate parameter values as input and select the highest scoring motifs across parameter settings automatically. Furthermore, Compo explicitly models fault-tolerant absence of motif instances in composite motifs. Finally, Compo is able to use several different approaches to pre-processing in the search procedure.

## Conclusion

The results on the benchmark suite show a very competitive quantitative performance for Compo using default parameters, in particular in cases where support across sequences may be utilized. In addition to this, Compo has some qualitatively advantageous properties. The intuitive parameters and discovery algorithm make the method relatively transparent, and the results are more easily interpretable compared to many other methods. The option of considering composite motif discovery as a multi-objective optimization problem allows users to spot higher-order trends in results and to postpone making trade-offs between objectives until after the search. Finally, with a general discovery algorithm and a relatively accessible Python source code, Compo lends itself to experimentation and further development.

## Methods

The main benchmark data set consists of all composite modules in the TransCompel database [25] that have at least five annotated instances. Details are given in Klepper *et al*. [24]. The prediction performance of Compo was compared against the methods Cister [12], Cluster-Buster [18], Stubb [31], ModuleSearcher [7], MScan [13], CMA [32], CisModule [9] and MCast [17]. The performance of each method was tested using PWMs compiled based on these binding sites (custom matrices version of benchmark). The robustness of predictions was tested by adding non-relevant (noise) motif matrices to the input data, as described in the original benchmark study [24]. Compo was also tested on data sets of liver-specific [27] and muscle-specific [26] gene sets taken from the literature, as well as a recent benchmark based on the REDfly database [28]. Visualizations of annotated binding sites in the muscle and liver data sets are given as Additional file 2 and 3, respectively.

Data on the other methods are taken from the original benchmark study [24], and are in general generated with default parameter settings. Since choosing the proper parameter values can sometimes prove crucial for performance, it was decided to provide the programs with a few general clues where applicable. The size of modules was specified as not exceeding 200 bp (300 bp in the muscle dataset). The modules were defined as consisting of exactly two single binding sites for different TFs in the TransCompel dataset, and possibly up to ten binding sites for four and five different TFs on the liver and muscle sets respectively. Furthermore, binding sites could potentially overlap, and the composition of the modules in liver and muscle sets was allowed to vary between sequences. As ModuleSearcher does not match the PWMs against the sequences itself, a program called MotifScanner was used as pre-processor for ModuleSearcher. Both of these programs were developed by the same group and are part of the Toucan suite of tools for regulatory sequence analysis [33].

The performance on benchmark data is given as the nucleotide-level correlation coefficient (nCC) from the comparison between predicted and known modules, as previously described e.g. in the benchmark studies of Tompa *et al*. [3] and Klepper *et al*. [24]. Here nTP, nFP, nTN and nFN represent true positive, false positive, true negative and false negative predictions at the nucleotide level.

$$nCC=\frac{nTP\cdot nTN-nFN\cdot nFP}{\sqrt{\left(nTP+nFN)(nTN+nFP)(nTP+nFP)(nTN+nFN\right)}}$$

In the benchmark suite compiled from the REDfly database, we followed the evaluation procedure defined in the article proposing the benchmark [28]. A collection of 53 PWMs accompanying the benchmark was used as single motif input for each data set. Here, Compo was compared against Stubb [31], MCD [28,34], D2z and CSAM [28].

## Availability and requirements

Compo is written in Python, and is freely available as source code under the GPL license at .

## Authors' contributions

GKS conceived the initial idea, devised the algorithms, implemented the method and drafted the main parts of the manuscript. OA contributed to the scientific content of the paper, formalized the machine learning perspective, drafted the section on problem definition and took part in writing on all parts of the manuscript. FD supervised and took part in all stages of the project.

## Supplementary Material

Additional file 1 **Additional formulas and experimentation details**. Further details on pruning of search space and computation of hit-probability, as well as details on the experimental setup. Click here for file

Additional file 2 **Binding sites in muscle data set**. A visualization of annotated binding sites in the muscle data set [26]. Click here for file

Additional file 3 **Binding sites in liver data set**. A visualization of annotated binding sites in the liver data set [27]. Click here for file
