# Supplementary material for: Compo: composite motif discovery using discrete models
Source: BMC Bioinformatics. 2008 Dec 8;9:527. doi: 10.1186/1471-2105-9-527 (PMC2614996; doi:10.1186/1471-2105-9-527)
Supplement: Additional file 1 — Additional formulas and experimentation details. Further details on pruning of search space and computation of hit-probability, as well as details on the experimental setup. [file 1471-2105-9-527-S1.pdf]

Supplementary Material for Sandve, Abul & Drabløs: Compo: composite motif discovery using discrete models

Enforcing distance constraints in support set computation

A central part of Compo is determination of the support set of a composite motif. In the basic set model without distance constraints, the support set of a composite motif is calculated simply as the intersection of the support sets of all component motifs. When using distance constraints, this becomes somewhat more complicated. As each single motif may have many hits, we can not simply compare a set of unique positions for each component motif. Instead, distance constraints can be checked either by sliding a window across a sequence of start- and end-positions or by enumerating motif hits for each component motif.

In a sliding window approach, the start and end positions of each hit for each component motif are sorted in increasing order. This sorted list can then be traversed from start to end by iteratively adding positions from the list to the front of a queue, and removing positions from the back of the queue when they have been passed by the sliding window (when a newly added position is further beyond a given position than the distance constraint). Each time a new position is added to the queue, a set of motifs having both start- and end-positions contained in the queue is updated. If the cardinality of this set is high enough (the number of component motifs, possibly minus the allowed number of motif misses), the composite motif has a hit in this sequence conforming to the distance constraints. This can be efficiently computed, with computation dominated by initial sorting of positions, or by the computation of the set of motifs contained in the queue at each time. As the sliding window may contain several hits for the same motif, computation of the set of contained motifs, and optionally checking for motif overlaps is however not straightforward.

In enumerating motif hits, the span between first and last position is calculated for each combination of hit positions for the component motifs. The minimum span is found and compared against the distance constraint. If single motif misses are allowed, the different ways of choosing a hit position for M out of the N component motifs are enumerated, with the span checked as before. If motif overlaps should not be allowed, this can be checked simply by testing testing each pair of motif hits. The enumeration of motif hits is exponential to the number of component motifs that have more than one hit. In practical cases, however, the number of component motifs is not very high. This approach can

handle motif misses and avoid overlaps, and can also be given a quite straightforward recursive implementation. We have therefore chosen to implement the enumeration approach.

Calculating hit-probability when allowing non-perfect matches

The hit-probability of a composite motif is calculated incrementally from the hit-probabilities of a base composite motif X and an added single motif y. Using the same vocabulary as in the main manuscript, the formula for incremental hit-probability can be derived as follows:

$$\begin{aligned}
P(Xy^1) &= \sum_{y=0,1} P(Xy^1, y) && (1)\\
&= P(Xy^1, y=1) + P(Xy^1, y=0) && (2)\\
&= P(Xy^1|y=1) \times P(y) + P(Xy^1|y=0) \times (1 - P(y)) && (3)\\
&= P(Xy^1|y=1) \times P(y) + P(X^0) \times (1 - P(y)) && (4)\\
&= P(X^1) \times P(y) + P(X^0) \times (1 - P(y)) && (5)\\
&= P(y) \times (P(X^1) - P(X^0)) - P(X^0) && (6)
\end{aligned}$$

In the formula, $P(y)$ and $P(y=1)$ are the same notations and therefore $P(y=0) = 1 - P(y)$ can be substituted. For simplicity of presentation the derivation is shown for the case of allowing 1 mismatch, but the derivation carries over directly to the general case of allowing up to n mismatches. Note that hits for the new motif y are assumed independent from hits for the motifs X in background, and note that H_X^{q-1} is a strict subset of H_X^q.

Calculating hit-probability under distance constraints

Hit-probability for the basic case is defined as the approximate probability that a motif has an instance in a random sequence, and is computed incrementally for composite motifs with a simple and efficient formula. The computation gets somewhat more complicated when the hits of all component motifs are required to be within a sequence window of a given length. Computing the approximate probability that a single motif hits in a single sequence window is done similarly to before: $Sw_z = 1 - (1 - p_{pos})^l$, where l is now length of the sequence window For composite motifs, this is as before computed incrementally:
$Sw_{X.z} = Sw_X * Sw_z$, where Sw_X and Sw_z now refer to probabilities for a single sequence window of base composite motif and new single motif respectively.

As the distance constraint refers to a sliding window, the composite motif can occur in one or more of several overlapping windows. We define the set of overlapping windows of length L in a sequence of length l as $\{w_i, for 0 <= i < l - L\}$. We also define the probability that a composite motif occurs in

a specific window, given that it does not occur in the preceding window (shift probability): $Ss = P(w_i | \not w_{i-1})$. For single motifs (with autocorrelation still ignored), this is simply the probability of the single motif having a hit at a specific position (p_{pos}). For composite motifs this is computed incrementally by the following formula:
$Ss_{X.z} = (Ss_X \& \not Sw_X \& Sw_z)|(Sw_X \& Ss_z \& \not Sw_z) = 1 - (1 - Ss_X \cdot (1 - Sw_X) \cdot Sw_z) \cdot (1 - Sw_X * Ss_z \cdot (1 - Sw_z))$

The (approximate) probability that a composite motif occurs in at least one of the overlapping windows, is then the probabilty of either occuring in the first window, or occuring in any other window, given that it did not occur in the preceding (overlapping) window:
$S = Sw_0 \| Ss_1 \| \ldots \| Ss_l = 1 - (1 - Sw) * (1 - Ss)^{(l-L)}$

Although the computation that consider distance constraints certainly alters the absolute values of significance, the ordering of motifs based on score will be roughly the same as if the hit-probability formula for the basic case was used. That is, for a specific distance constraint value, if a motif A scores higher than motif B using the significance computation described in this section, it will usually also score higher with the simple formula given for the basic case (although exceptions to this can be easily constructed). As motif scoring is only a rough approximation to the biological case, we do not see these small inaccuracies as problematic. This means that we can use the simple and computationally efficient formula to rank motifs even when we apply a single distance constraint. If we want to compare composite motifs across different distance constraints, we make use of the significance evaluation described here. For each value of distance constraint we can then use the simple formula to evaluate and rank motifs during search. Afterwards, the best scoring candidate motifs for each distance constraint value are compared using the significance formulas described in this section. Finally, the best scoring composite motif across distance constraint values is returned.

It is quite straightforward to combine constraints on distance described here with allowing motif misses as described in a previous section. The changes to the basic formulas that were needed for each extension are basically combined. This has been done in our implementation which supports the discovery of composite motifs with distance constraints while also allowing motif misses. The details of this can be inspected in the freely available Python source code.

Branch-and-bound approach

A branch-and-bound approach is used to prune the search tree of composite motifs. This requires efficient computation of a reasonably tight bound for the score in a subtree.

As we do not consider any ordering between components, we choose to only traverse composite motifs with increasing motif index on added single motifs. This avoids exploring several permutations of single motifs that corresponds to the same unordered combination. By initially sorting the list of single motifs

according to increasing hit-probability, we know that a newly added single motifs will always have equal or higher hit-probability compared to the last added single motif. This allows computing a bound on hit-probability for the subtree efficiently. As the support of a composite motif can never increase when adding new components (for the same number of allowed misses), the current support of a composite motif is also a bound on the support on any composite motif in the subtree.

We compute bounds in a general way by iteratively adding optimistic virtual single motifs up to the maximum allowed number of components. If this maximum number is reached without any of the virtual composite motifs exceeding the bound, the original composite motif is pruned. An optimistic motif is simply a virtual motif with significance equal to the computed upper bound, and hits in all sequences. For the basic case this simply amounts to a bound on support equal to the current support, and a bound on hit-probability that is the hit-probability of the last added single motif raised to the number of additional components allowed, multiplied by the current composite motif hit-probability.

Computational efficiency and numerical concerns

As the number of single motifs might typically be much higher than the number of sequences (especially if discrete maximal motifs are used, as output by e.g. Teiresias.), we have implemented item set mining algorithms that suites this. We enumerate combinations of motifs and incrementally update the support set of Composite motifs. For very large number of motifs, we have made an implementation in C++ using efficient bitstring-operations directly on bitstrings representing the support set [1]. As both support set and hit-probability might be time-consuming to compute from ground up for each composite motif considered, we also use simple operations that incrementally compute these values from parents as the search tree is traversed in a depth-first manner.

For very long and specific motifs, the hit-probability P_x will typically be very low. If motif significance is used as combined measure for interestingness, the values may become very small and introduce numerical problems in calculations. Log-values may therefore be used to represent hit-probability and motif significance. In the basic incremental calculations of hit-probability, multiplications are replaced by additions, also making the calculations more computationally efficient. When allowing non-perfect composite motif matches there are some additions in the original incremental formulas, making the calculations based on log-values slightly more complex and time consuming. As default we have only used log-values intermediately in motif significance calculations in order to make the source code more readable. In the C++ implementation that make use of bitstring calculations for support-set, we have also used log-values throughout to further increase the computational efficiency.

Expected binding site motifs

In the TransCompel data set the following pairs of transcription factor binding site motifs are represented (as individual composite motif data sets): AP1-Ets, AP1-NFkappaB, Ebox-Ets, IRF-NFkappaB, PU1-IRF, AP1-NFAT, CEBP-NFkappaB, Ets-AML, NFkappaB-HMGIY, Sp1-Ets

The muscle data set contain the following motifs: Mef2, Myf, Sp1, SRF and TEF

The liver data set contain the following motifs: HNF-1, HNF-3, HNF-4 and CEBP

Detailed locations of annotated binding sites are available for download at: http://tare.medisin.ntnu.no/composite/composite.php

Experimental details

For the main TransCompel-based benchmark, Compo was run in default mode with automatic selection of parameter values from a list of discrete possibilities. To avoid very long running times when large lists of single motifs were supplied, the search space of Compo was limited to ten million candidate composite motifs. Compo then for each data set automatically set the maximum number of composite motif components that would not lead to exceeding the search space limit based on the number of supplied input motifs. The distance window restriction were allowed to range from 50 to 200 base pairs long in three discrete steps, and the hit factor used in determining single motif hits were allowed to range from 1 to 2 in three steps. A randomly selected set of upstream regions from the human genome were used as background. Motifs across all parameter settings were ranked according to their computed p-value, and the composite motif with lowest p-value returned.

As the muscle and liver data sets have more heterogeneous TF regulation, we there had each sequence considered in isolation as the main run. We also provided results when considering support across sequences, and then for one variant where all component motifs were required to hit and one variant where we allowed hits for up to two components to be missing from a composite motif occurrence. In all muscle and liver runs we allowed a composite motif to contain up to 8 components, we allowed a composite motif to contain several instances of the same single motif, and allowed Compo to make several predictions per sequence as long as each predicted composite motif had a significance value (p-value) below 0.05.

For the Drosophila benchmark, Compo was run with TP-factor ranging from 1 to 4 in three steps, with a maximum of 6 components of composite motifs, with up to 2 components missing in each composite motif instance, and with a distance window ranging from 400 to 1000. Composite motifs predicted by Compo will not always have instances in all input sequences. The evaluation scripts accompanying the benchmark required predictions to be made for every sequence in order to work correctly, and we did not want to change these external scripts. To solve this problem, we therefore had Compo fill in a fixed predicted

location at position 1000 in the sequences where its predicted composite motifs did not have any instances.

Detailed predictions

The following tables shows the predicted motifs in the TransCompel, muscle and liver benchmark suites. In the TransCompel suite, each sequence in a data set have binding sites for the same two motifs, and predictions are therefore given for each data set. Also, results are given at different levels of added noise motifs. For the muscle and liver data sets, the individual sequences has binding sites for different sets of motifs, and predictions are therefore given for each sequence of these data sets. For all sequences of the liver data set the motifs CEBP,HNF-1,HNF-3 and HNF-4 are given as input. For all sequences of the muscle data set Mef2, Myf, Sp1, SRF and TEF are given as input.

TransCompel benchmark suite, noise level 0%

Dataset	Motif IDs
AP1-Ets	AP1
AP1-NFAT	AP1,NFAT
AP1-NFkappaB	NFkappaB
CEBP-NFkappaB	NFkappaB,CEBP
Ebox-Ets	Ets,Ebox
Ets-AML	Ets,AML
IRF-NFkappaB	NFkappaB,IRF
NFkappaB-HMGIY	NFkappaB
PU1-IRF	IRF
Sp1-Ets	Sp1

TransCompel benchmark suite, noise level 50%

Dataset	Motif IDs
AP1-Ets	AP1
AP1-NFAT	AP1,NFAT
AP1-NFkappaB	NFkappaB
CEBP-NFkappaB	NFkappaB,CEBP
Ebox-Ets	Ets,Ebox
Ets-AML	Ets,AML
IRF-NFkappaB	NFkappaB,IRF
NFkappaB-HMGIY	NFkappaB
PU1-IRF	IRF
Sp1-Ets	Sp1

TransCompel benchmark suite, noise level 90%

Dataset	Motif IDs
AP1-Ets	AP1
AP1-NFAT	M00194,NFAT
AP1-NFkappaB	NFkappaB,M00633
CEBP-NFkappaB	NFkappaB,CEBP
Ebox-Ets	M00799,M00967
Ets-AML	Ets,AML
IRF-NFkappaB	NFkappaB,IRF
NFkappaB-HMGIY	NFkappaB
PU1-IRF	IRF
Sp1-Ets	M00660,Ets

TransCompel benchmark suite, noise level 95%

Dataset	Motif IDs
AP1-Ets	AP1
AP1-NFAT	M00933,NFAT
AP1-NFkappaB	NFkappaB
CEBP-NFkappaB	NFkappaB,CEBP
Ebox-Ets	Ebox,M00644
Ets-AML	Ets,M00929
IRF-NFkappaB	NFkappaB,IRF
NFkappaB-HMGIY	NFkappaB,M00396
PU1-IRF	M00648,IRF
Sp1-Ets	Ets,M00945

TransCompel benchmark suite, noise level 99%

Dataset	Motif IDs
AP1-Ets	M00117,AP1
AP1-NFAT	M00144,M00208
AP1-NFkappaB	M00821,NFkappaB
CEBP-NFkappaB	NFkappaB,CEBP
Ebox-Ets	M00344,M00967
Ets-AML	M01080,M01030
IRF-NFkappaB	NFkappaB,IRF
NFkappaB-HMGIY	M00252,NFkappaB
PU1-IRF	M00687,IRF
Sp1-Ets	M00803,M00428

Muscle benchmark suite

Sequence	Motif IDs
J04699	sp1,mef2,tef,myf
J04971	sp1,srf,tef,mef2,myf
K01464	srf,myf
L21905	sp1,srf,tef,mef2,myf
M13483	sp1,srf,tef
M13631	sp1,srf
M20543	sp1,srf
M21390	sp1,srf,mef2,myf
M22381	sp1,srf,myf
M57905	sp1,srf,tef,myf
M62404	sp1,srf,mef2,tef,myf
M63391	sp1,srf,tef,myf
M95800	sp1,srf,mef2,tef,myf
U02285	sp1,srf
U18131	sp1
V01218	srf,myf
X05632	sp1,srf,mef2,tef,myf
X12971	sp1,srf,tef,myf
X14726	srf,tef,mef2,myf
X62155	sp1,srf,mef2,myf
X67686	sp1,srf
X73887	sp1,srf,mef2,tef,myf

Liver benchmark suite

Sequence	Motif IDs
AF033857	HNF-4,HNF-3,CEBP,HNF-1
AF051355	HNF-4,HNF-3,CEBP,HNF-1
AF236668	HNF-3,CEBP,HNF-1
L09674	HNF-4,CEBP,HNF-1
L13460	HNF-4,HNF-3,HNF-1
M15657	HNF-4,HNF-3,HNF-1
M19524	HNF-4,HNF-3,HNF-1
M29301	HNF-4,HNF-3,CEBP,HNF-1
M60197	HNF-3,CEBP,HNF-1
S85346	HNF-3,CEBP,HNF-1
U47685	HNF-3,HNF-1
X16152	CEBP

References

1. Sandve GK, Drabløs F: **Generalized Composite Motif Discovery**. In *7th Int. Conf. on Knowledge-Based Intelligent Information and Engineering Systems, KES*, *Volume 3683 of* LNCS/LNAI, Springer-Verlag 2005:763–769.
