# Supplementary material for: Compo: composite motif discovery using discrete models
Source: BMC Bioinformatics. 2008 Dec 8;9:527. doi: 10.1186/1471-2105-9-527 (PMC2614996; doi:10.1186/1471-2105-9-527)
Supplement: Additional file 2 — Binding sites in muscle data set. A visualization of annotated binding sites in the muscle data set [26]. [file 1471-2105-9-527-S2.pdf]

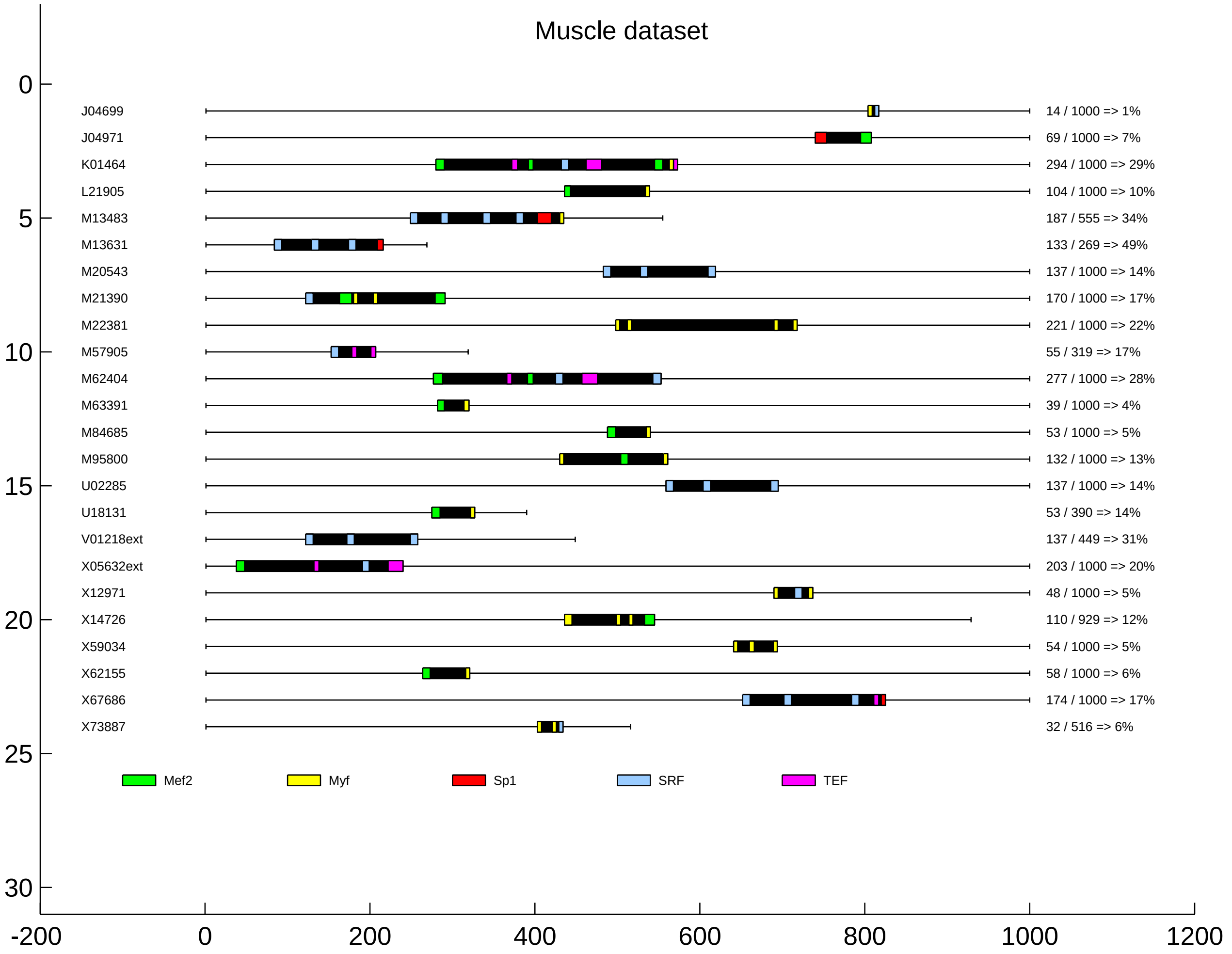
Muscle dataset
J04699
J04971
K01464
L21905
M13483
M13631
M20543
M21390
M22381
M57905
M62404
M63391
M84685
M95800
U02285
U18131
V01218ext
X05632ext
X12971
X14726
X59034
X62155
X67686
X73887
14 / 1000 => 1%
69 / 1000 => 7%
294 / 1000 => 29%
104 / 1000 => 10%
187 / 555 => 34%
133 / 269 => 49%
137 / 1000 => 14%
170 / 1000 => 17%
221 / 1000 => 22%
55 / 319 => 17%
277 / 1000 => 28%
39 / 1000 => 4%
53 / 1000 => 5%
132 / 1000 => 13%
137 / 1000 => 14%
53 / 390 => 14%
137 / 449 => 31%
203 / 1000 => 20%
48 / 1000 => 5%
110 / 929 => 12%
54 / 1000 => 5%
58 / 1000 => 6%
174 / 1000 => 17%
32 / 516 => 6%
Mef2
Myf
Sp1
SRF
TEF
0
5
10
15
20
25
30
-200
0
200
400
600
800
1000
1200

Selected modules in muscle sequences

	Mef2 (A/T-rich)	Myf (MyoD, E-box)	Sp1 (GC-box)	SRF (CArG)	TEF (M-CAT)
MUS01: X59034		**3**			
MUS03: M22381		**4**			
MUS09: M13483		**1**	**1**	**4**	
MUS10: M21390	**2**	**2**		**1**	
MUS12: M63391	**1**	**1**			
MUS15: U18131	**1**	**1**			
MUS16: M84685	**1**	**1**			
MUS18: X62155	**1**	**1**			
MUS19: M95800	**1**	**2**			
MUS21: X14726	**1**	**3**			
MUS22: X12971		**2**		**1**	
MUS23: J04971	**1**		**1**		
MUS24: L21905	**1**		**1**		
MUS27: M57905				**1**	**2**
MUS28: X73887		**2**			**1**
MUS29: J04699		**1**			**1**
MUS34: X05632ext	**1**			**1**	**2**
MUS36: M62404	**2**			**2**	**2**
MUS37: K01464	**3**	**1**		**1**	**3**
MUS38: V01218ext				**3**	
MUS39: U02285				**3**	
MUS41: M20543				**3**	
MUS42: M13631				**3**	**1**
MUS43: X67686			**1**	**3**	**1**
Sum	**16**	**25**	**4**	**26**	**13**

24 modules in 24 sequences
5 different motifs
84 TF binding sites

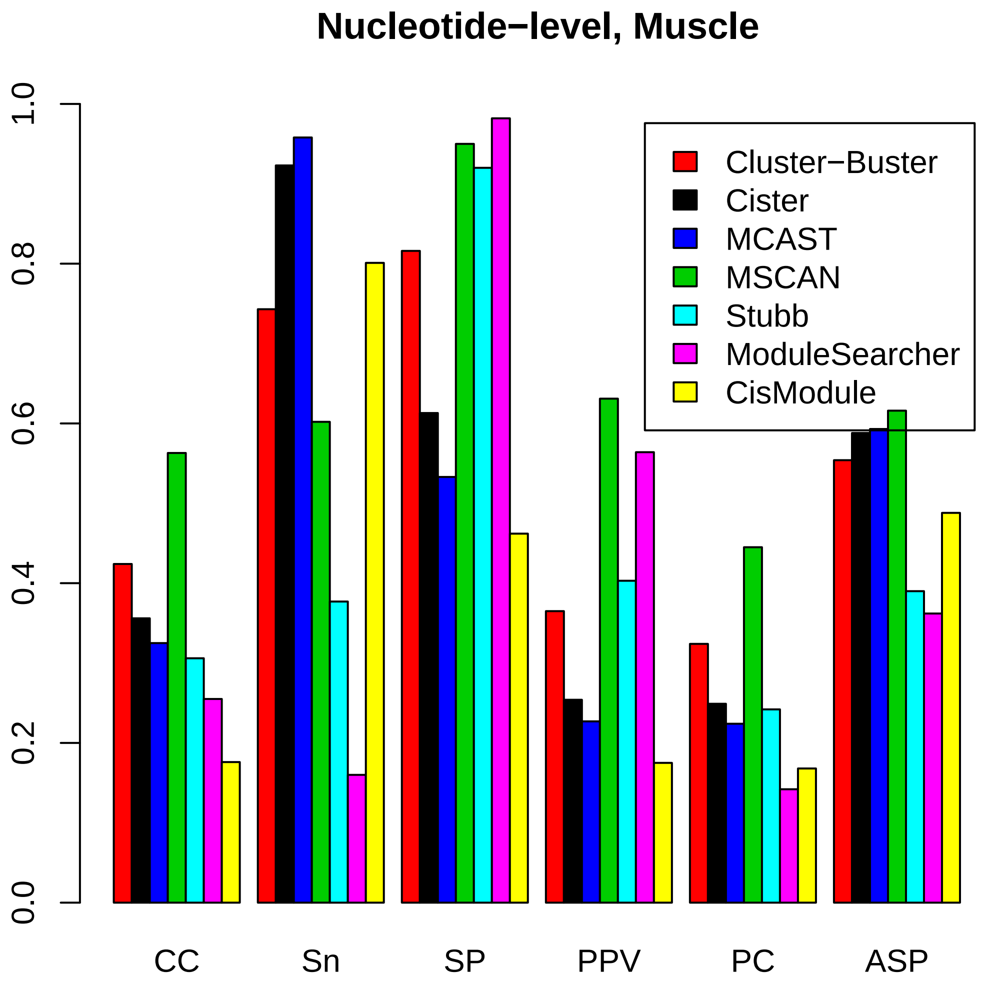

Nucleotide−level, Muscle
0.0
0.2
0.4
0.6
0.8
1.0
Cluster−Buster
Cister
MCAST
MSCAN
Stubb
ModuleSearcher
CisModule
CC
Sn
SP
PPV
PC
ASP

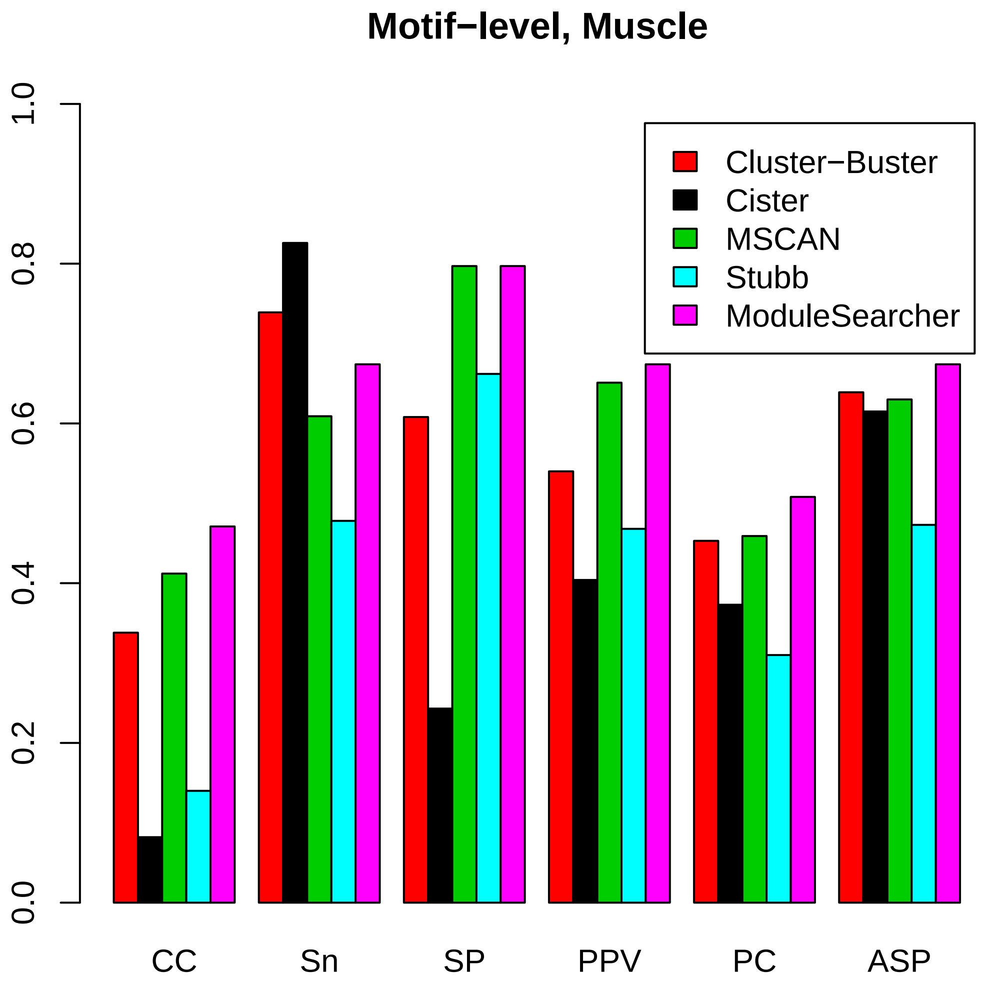
Motif−level, Muscle
Cluster−Buster
Cister
MSCAN
Stubb
ModuleSearcher
0.0
0.2
0.4
0.6
0.8
1.0
CC
Sn
SP
PPV
PC
ASP
