# Supplementary material for: Compo: composite motif discovery using discrete models
Source: BMC Bioinformatics. 2008 Dec 8;9:527. doi: 10.1186/1471-2105-9-527 (PMC2614996; doi:10.1186/1471-2105-9-527)
Supplement: Additional file 3 — Binding sites in liver data set. A visualization of annotated binding sites in the liver data set [27]. [file 1471-2105-9-527-S3.pdf]

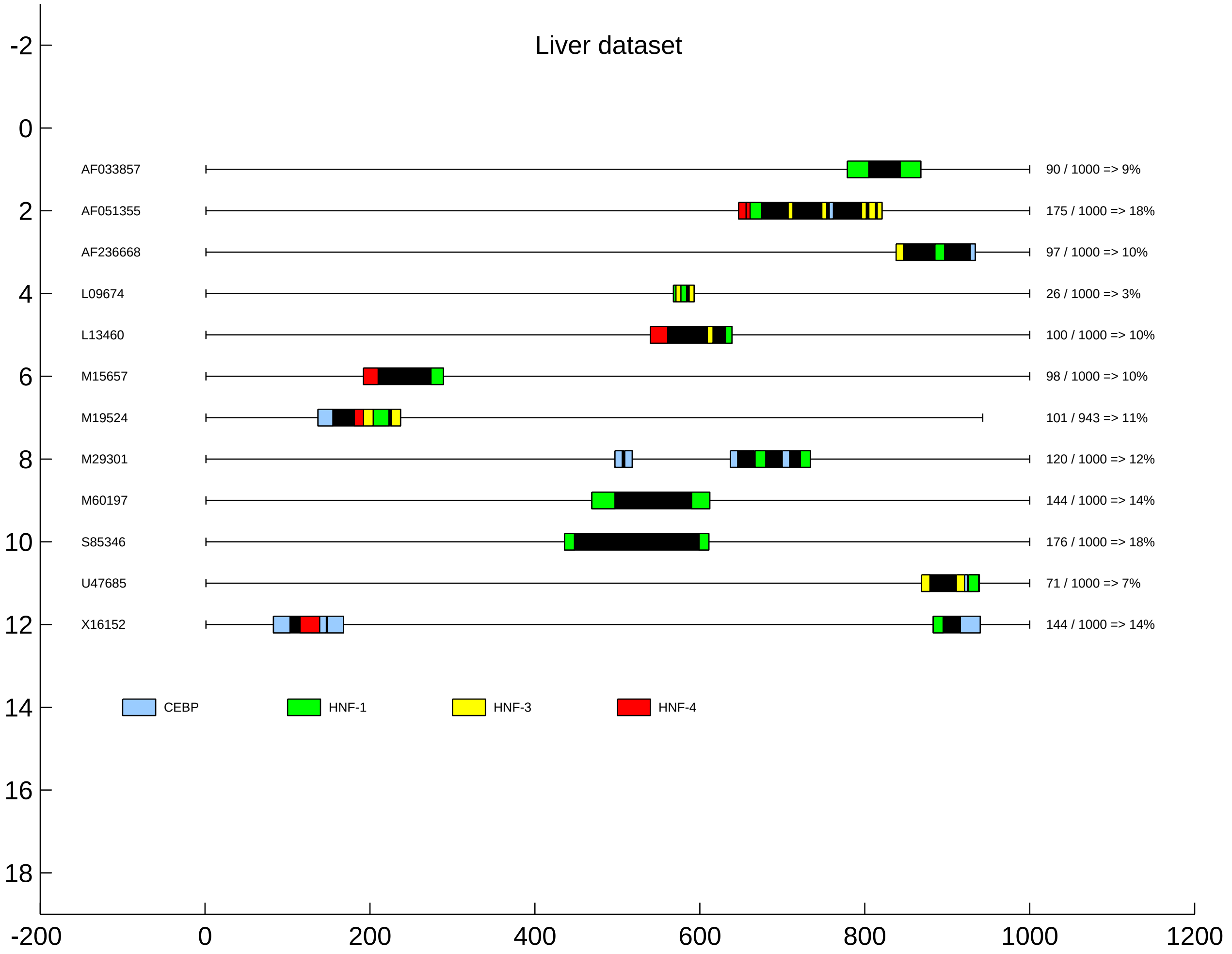
Liver dataset
AF033857
AF051355
AF236668
L09674
L13460
M15657
M19524
M29301
M60197
S85346
U47685
X16152
90 / 1000 => 9%
175 / 1000 => 18%
97 / 1000 => 10%
26 / 1000 => 3%
100 / 1000 => 10%
98 / 1000 => 10%
101 / 943 => 11%
120 / 1000 => 12%
144 / 1000 => 14%
176 / 1000 => 18%
71 / 1000 => 7%
144 / 1000 => 14%
CEBP
HNF-1
HNF-3
HNF-4
-2
0
2
4
6
8
10
12
14
16
18
-200
0
200
400
600
800
1000
1200

Modules

	HNF-1	HNF-3	HNF-4	CEBP
AF033857	**2**			
AF051355	**1**	**5**	**2**	**1**
AF236668	**1**	**1**	**0**	**1**
L09674	**1**	**2**		
L13460	**1**	**1**	**1**	
M15657	**1**		**1**	
M19524	**1**	**2**	**1**	**2**
M29301 (a)				**2**
M29301 (b)	**2**			**3**
M60197	**2**			
S85346	**2**			**1**
U47685	**2**	**2**		**1**
X16152 (a)	**1**			**1**
X16152 (b)			**1**	**3**

14 modules in 12 sequences
4 different motifs
51 binding sites

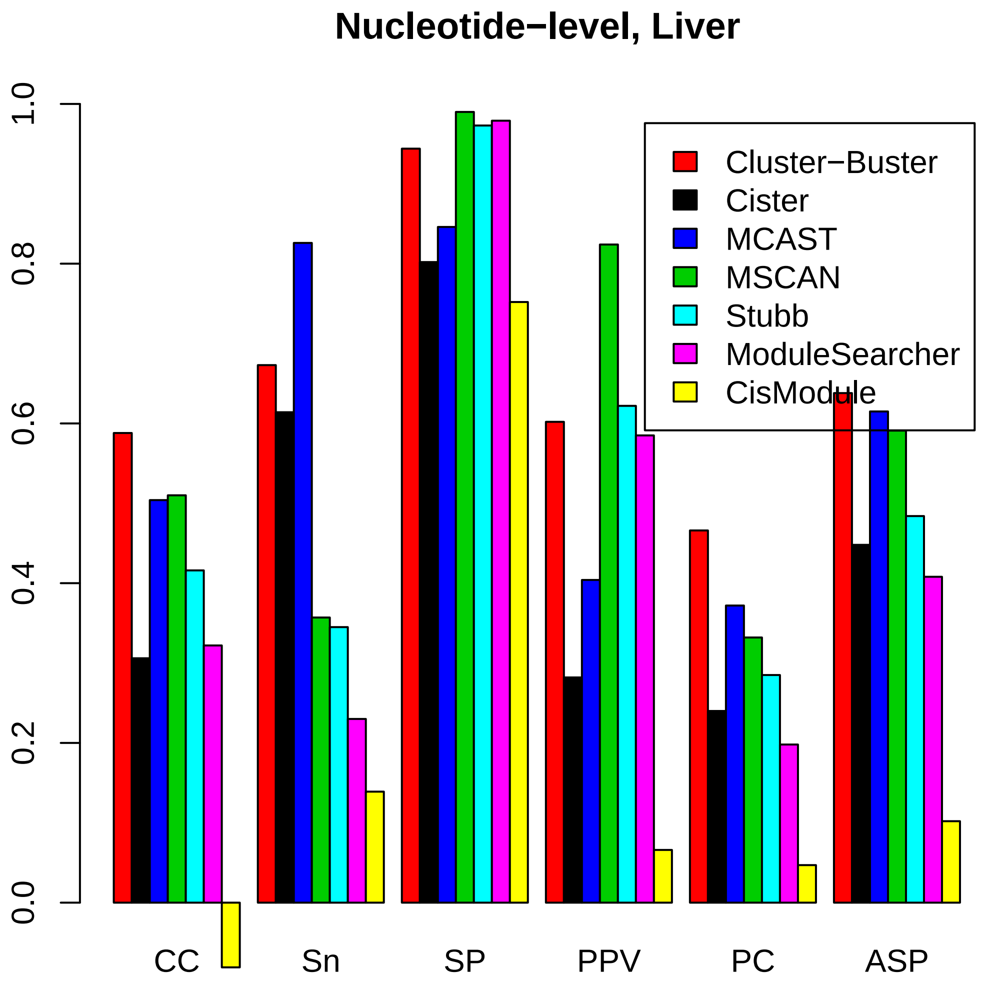

Nucleotide−level, Liver
0.0
0.2
0.4
0.6
0.8
1.0
Cluster−Buster
Cister
MCAST
MSCAN
Stubb
ModuleSearcher
CisModule
CC
Sn
SP
PPV
PC
ASP

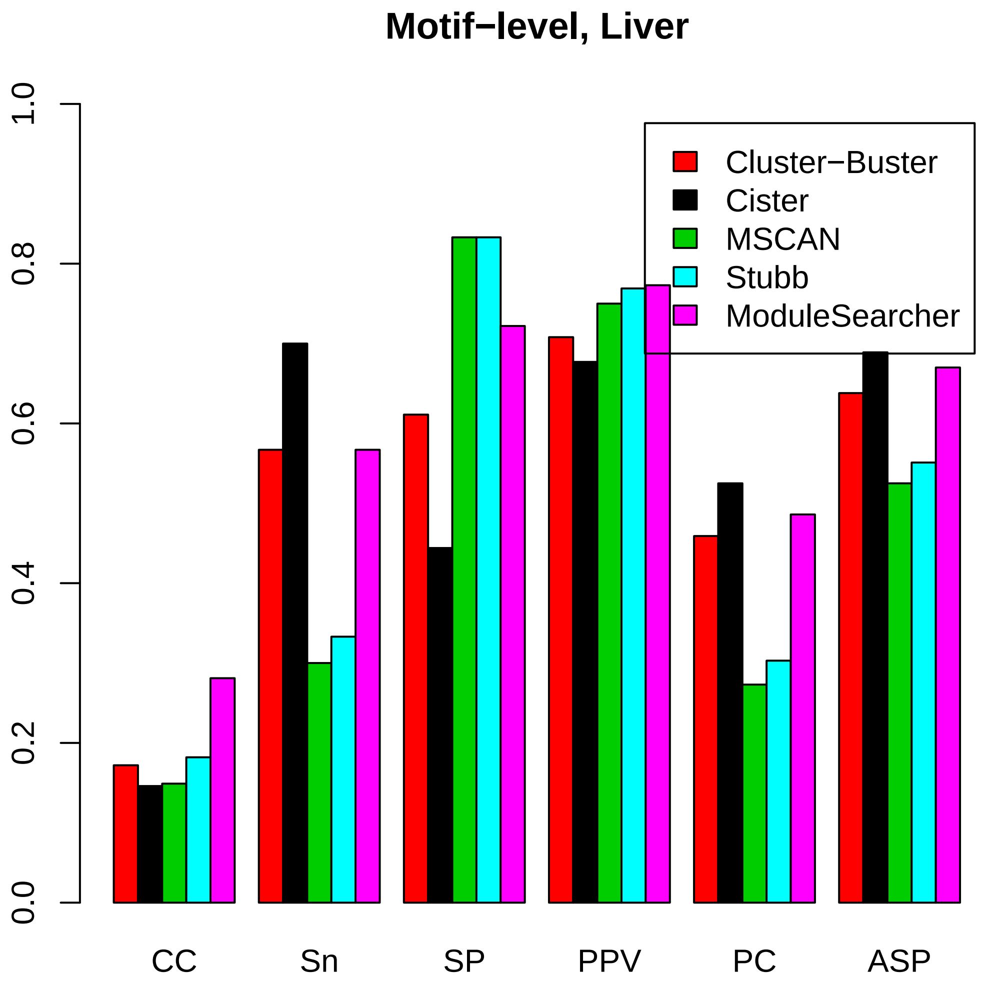
Motif−level, Liver
0.0
0.2
0.4
0.6
0.8
1.0
Cluster−Buster
Cister
MSCAN
Stubb
ModuleSearcher
CC
Sn
SP
PPV
PC
ASP
